# Land-use change and rodent-borne diseases: hazards on the shared socioeconomic pathways

**DOI:** 10.1098/rstb.2020.0362

**Published:** 2021-11-08

**Authors:** Gabriel E. García-Peña, André V. Rubio, Hugo Mendoza, Miguel Fernández, Matthew T. Milholland, A. Alonso Aguirre, Gerardo Suzán, Carlos Zambrana-Torrelio

**Affiliations:** ^1^ Centro de Ciencias de la Complejidad, Universidad Nacional Autónoma de México, Ciudad de México, Mexico; ^2^ Departamento de Etología, Fauna Silvestre y Animales de Laboratorio, Facultad de Medicina Veterinaria y Zootecnia, Universidad Nacional Autónoma de México, Ciudad de México, Mexico; ^3^ Departamento de Ciencias Biológicas Animales, Facultad de Ciencias Veterinarias y Pecuarias, Universidad de Chile, Santiago, Chile; ^4^ NatureServe, Arlington, VA, USA; ^5^ Department of Environmental Science and Policy, George Mason University, Fairfax, VA, USA; ^6^ University of Maryland, AGNR-Environmental Science and Technology, College Park, MD, USA; ^7^ EcoHealth Alliance, New York, NY, USA

**Keywords:** disease risk, emerging zoonotic diseases, environmental change, land-use scenarios, Rodentia

## Abstract

Land-use change has a direct impact on species survival and reproduction, altering their spatio-temporal distributions. It acts as a selective force that favours the abundance and diversity of reservoir hosts and affects host–pathogen dynamics and prevalence. This has led to land-use change being a significant driver of infectious diseases emergence. Here, we predict the presence of rodent taxa and map the zoonotic hazard (potential sources of harm) from rodent-borne diseases in the short and long term (2025 and 2050). The study considers three different land-use scenarios based on the shared socioeconomic pathways narratives (SSPs): sustainable (SSP1-Representative Concentration Pathway (RCP) 2.6), fossil-fuelled development (SSP5-RCP 8.5) and deepening inequality (SSP4-RCP 6.0). We found that cropland expansion into forest and pasture may increase zoonotic hazards in areas with high rodent-species diversity. Nevertheless, a future sustainable scenario may not always reduce hazards. All scenarios presented high heterogeneity in zoonotic hazard, with high-income countries having the lowest hazard range. The SSPs narratives suggest that opening borders and reducing cropland expansion are critical to mitigate current and future zoonotic hazards globally, particularly in middle- and low-income economies. Our study advances previous efforts to anticipate the emergence of zoonotic diseases by integrating past, present and future information to guide surveillance and mitigation of zoonotic hazards at the regional and local scale.

This article is part of the theme issue ‘Infectious disease macroecology: parasite diversity and dynamics across the globe’.

## 1. Introduction

Land-use change—the conversion of native vegetation to anthropogenic habitats—is a significant driver of biodiversity loss and ecosystem degradation worldwide [1] and has also been credited with the emergence of zoonotic infectious diseases [2–4]. Previous work suggests that land-use change may increase interactions among wildlife, domestic and synanthropic animals, and humans. These interactions favour cross-species transmission of pathogens, promoting disease emergence [5–8]. Although the mechanisms behind disease emergence and land-use change are not well understood, land-use change may cause local extinction of species and modify the abundance of hosts and the composition and structure of communities, thus changing pathogen transmission dynamics [7,9,10]. Similarly, changes in host communities can restructure host–pathogen associations, altering pathogen abundance and richness and reshaping the pathogen communities to which humans are exposed [9–12]. In general, these processes increase pathogen prevalence in hosts adapted to human-dominated landscapes [13].

Changes in land-use patterns that, in turn, impact the ecological interactions of the different local communities increase the probability of emerging zoonotic diseases, such as Ebola, Nipah virus, Japanese encephalitis, Lyme disease, plague and hantavirus cardiopulmonary syndrome (HPS) [14–17]. As evidenced by the current COVID-19 pandemic, zoonotic infectious diseases can have disastrous effects on the world economy and public health [18–20]. Hence, human adaptation and resilience require surveillance mechanisms and strategic control of zoonotic diseases, particularly in areas where the risks are high.

Future land-use scenarios are captured by the narratives of the shared socioeconomic pathways (SSPs), which predict future land-use change based on the demands of food and energy of a growing population [21]. The implications that these pathways may have on the disease risk emergence has not been explicitly considered. Here, we define risk as a composite metric of hazard (presence of hosts, vectors and microbes that may cause harm), human exposure (contact with hazard) and vulnerability (likelihood of harm given exposure). However, this approach has rarely been applied to define areas of epidemiological concern (but see [22]).

Previous studies predicted critical areas for disease emergence [3,23,24]. However, these studies did not incorporate the effects of land-use change on the spatial distribution of host species, a key element to find hotspots of emergence and transmission of infectious diseases. To contribute to this effort, we modelled and mapped the zoonotic hazard of rodent-borne diseases in the future 2050, accounting for the effects of land-use change in the geographical distribution of rodents. A zoonotic hazard is defined as the additive probabilities of finding rodent species in a location (owing to its land use), times the species-specific likelihood that the species carry pathogens harmful to humans according to their reservoir status, using the ‘equilibrium prevalence’, an epidemiological metric based on species' ecological traits [25] (see details in Material and methods).

## 2. Material and methods

### (a) Study taxa

Rodents (Rodentia) are the most ubiquitous mammals globally with approximately 2277 species, accounting for more than 42% of the global mammal biodiversity [26]. Currently, they are distributed on all continents and ecosystems except Antarctica. Rodents are among the most important hosts of infectious diseases worldwide [27,28], being associated with more than 80 zoonotic diseases [27]. Major rodent-borne diseases include HPS, haemorrhagic fever with renal syndrome, plague and leptospirosis [29]. Recent studies have shown that land-use change has a differential effect on rodent communities, where rodent reservoirs are favoured, generally increasing their richness or abundance as a result of anthropogenic disturbance in natural ecosystems and habitat conversion into agricultural lands [10,30]. This is explained by the fact that most rodent reservoirs are r-selected species, i.e. earlier sexual maturity and higher reproductive rate [31,32], which make them highly resilient to disturbances and able to colonize different anthropogenic settings [10,30].

### (b) Data

#### (i) Geographical distribution of rodents

We based our study on rodent maps produced by the International Union for Conservation of Nature [33] that provide geographical distribution for all vertebrates, describing the extent of occurrence for each species. Extent of occurrence quantifies the area contained within a polygon around sites where the species has been observed. This measure may exclude some discontinuities or disjunctions in the species’ spatial distribution, including large areas of unsuitable habitat. Although this is an extremely valuable product used extensively in conservation and biodiversity strategies, these maps are at best coarse approximations to the actual distribution of species. Species distributions were delineated under historical scenarios or potential conditions without including changes in their habitat suitability to land-use change or other anthropogenic factors, limiting its applications to other fields.

In this study, the distribution of rodents was refined using a novel approach that follows the trajectory of land-use change over time to model the probability of a species occurring at a given location. This temporal trajectory allowed us to project the distribution in space within the known distribution of each species, accounting for the land cover condition at the time of data collection. We used georeferenced presence data for all rodent species currently available in the Global Biodiversity Information Facility (GBIF) (doi:10.15468/dl.pqwhfw). We only considered records reported from specimens deposited in natural history museums, thus reducing taxonomic inconsistencies and misidentifications. Additionally, the presence data that coincided with centroids of countries, cities, museums, universities, institutions and GBIF headquarters were excluded. In total, 1203 species of rodents were analysed represented by 1 648 549 specimens preserved in museum collections worldwide. For each of these rodent species, we modelled presence probability given land-use data (see §2c on Data analysis).

#### (ii) Rodent reservoir status

We obtained data on the rodent capacity of transmitting zoonotic pathogens from publicly available data published by Han *et al*. [25]. These data are drawn from data mining, machine learning and susceptible-infectious-recovered models to estimate an ‘equilibrium prevalence’ for each rodent species. Equilibrium prevalence is an epidemiological metric that estimates the risk of human exposure to a directly transmitted pathogen based on the species' ecological traits as indicators of transmission risk rather than the actual prevalence of a pathogen [25]. In principle, fast-lived species with large litter size, early sexual maturity and high reproduction rates tend to host more zoonotic pathogens and may represent a significant source of exposure to zoonosis than those with slow-lived features, e.g. small litter size, late sexual maturity, low reproduction rate [31,34]. Equilibrium prevalence estimations do not include information on the degree of interaction with humans, and thus we used this information as a metric of hazard. In this study, we distinguished between risks and hazards [22,35,36]. While hazards are potential sources of harm, risks are the likelihood of an adverse event caused by the hazards and their severity, e.g. the number of people affected. Supplementary information provides details on how equilibrium prevalence was determined by Han *et al*. [25].

#### (iii) Land-use change

Associations between land-use change and rodent species were found by intersecting historical data on land use and the presence of rodents. Data from the History of the Global Environment database (HYDE) were used as surrogates of historical land-use [37] and the World Climate Research Program Coupled Model Intercomparison Project-CMIP6 [38], integrated with the Land-Use Harmonization (LUH2) project [21]. The LUH2 project integrates geographical data describing the percentage of 12 land-use states within cells of 0.25 arc degrees: these states include primary forest land; non-forested primary land; potentially forested secondary land; potentially non-forested secondary land; managed pasture; rangeland; urban land; annual crops (e.g. rice and sugarcane); perennial crops (e.g. *Miscanthus*) and nitrogen-fixing crops (e.g. alfalfa).

In addition to historical data on land-use change, LUH2 also provides different future land-use change scenarios following the SSPs, including (i) a sustainable development scenario (SSP1); (ii) a world with closed borders and deepening inequality (SSP4); and (iii) a future based on fossil fuel and trading (SSP5) [39–42]. SSP1 describes an environmentally aware humanity expected to achieve a maximum warming of 2°C. Socioeconomic and climate policies are in place to reduce agricultural land substantially, forested areas increase, and large regions are used to produce bioenergy [43,44]. By 2100, the sustainable scenario leads to a Representative Concentration Pathway of 2.6 radiative forcing (RCPs) [45]. By contrast, the SSP4 scenario describes a world in which technology is paramount to reduce the impacts of population growth. High-income economies are likely to fulfill the energy and food demands by intensifying livestock production, while the poorest countries must sustain their demanding population growth by transforming land into crop fields. Globally, borders are not open as in the SSP5 (see below), leading to higher inequalities between nations and big challenges for adaptation [41]. Finally, the SSP5 scenario describes a fossil-fuelled future, and assumes that population growth is governed by fossil fuel development, and increased food and energy demands are fulfilled by transforming pastures and forest into crop fields, open borders and international trade [42,46,47]. This scenario leads to an RCP of 8.5 in 2100 and entails big challenges for mitigation policies.

With these insightful data at hand, the near future of a sustainable scenario (SSP1) in 2025 was considered as a baseline to compare the future expected in 2050 under the scenarios: SSP1, SSP4 and SSP5.

### (c) Data analysis

**Figure 1.**
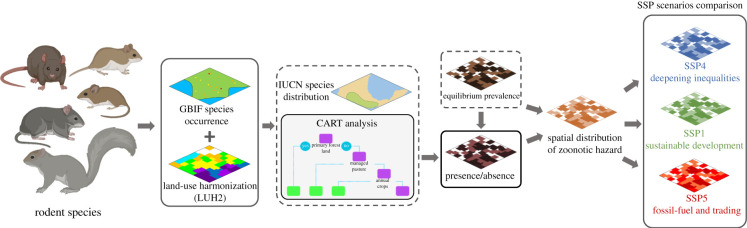
Conceptual model describing the data analysis strategy. Historical data on land use was associated with the capture date of the specimens of rodents (Rodentia) preserved in museums and reported in the Global Biodiversity Information Facility (GBIF). We used these data to train a model CART that predicts species presence probability within the species' distribution area, considering land use. The cumulative probabilities of species’ presence times the equilibrium prevalence of each species give an index of zoonotic hazard at each location. Zoonotic hazard was then projected to different land-use scenarios following the shared socioeconomic pathways (SSPs) narratives.

Analysis is presented in figure 1 and is explained in chronological order, in the following subsections.

#### (i) Classification and regression tree analysis: land-use change and species presence

All the rodents (Rodentia) in the world were considered the sample universe of our analyses, from which 1 648 549 specimens have been reported in GBIF with dates and coordinates of capture, and are preserved and classified in museum collections (doi:10.15468/dl.pqwhfw). For each of these specimens, we matched the historical land-use data at the time the rodent specimens were captured. This dataset allowed us to compare the land-use variables that predict the presence of a rodent species to variables associated with a random sample of any other rodent species in the GBIF dataset. Species with at least 10 specimens in the database were included as an arbitrary threshold that permits statistical inference.

Classification and regression trees (CART) and recursive partitioning are a machine learning technique that allows finding hierarchical relationships between predictors [48–50]. Among all the land-use variables (predictors), the one variable that best separated the two classes (presence or absence) and minimized a cross-validation error was used to split the presence data into two subsets. The same analysis was then performed on each presence subset which was split by the next best predictor; and so on recursively.

The procedure was repeated until the less complex tree with the least cross-validation error was found to avoid over parameterization. The recursive trees ranged from a cross-validation error of 0.5–1, with a median of 0.9 (electronic supplementary material, table S1). Finally, using the CART model fitted and trained with historical data, the probability of presence of each species was predicted in the future under different scenarios of land-use change: SSP1, SSP4 and SSP5.

#### (ii) Geographical cell analysis to estimate hazard

Map Algebra was used to perform cell by cell arithmetic [51]. For the future land-use scenarios, zoonotic hazard was estimated by multiplying the presence probability of each rodent species (*i*) at each geographical cell (*x*) times the equilibrium prevalence (*Θ*). Note that the equilibrium prevalence is an attribute of a species that does not change in time. This assumption is plausible as the prevalence is based on the life-history traits of each species. Hence, we estimated zoonotic hazard at each geographical cell_*x*, of the future scenarios, by using equation (2.1), where the presence probabilities of species *i* was added as estimated by CART models on each land-use change scenario. In other words, zoonotic hazard at a geographical cell was the addition of presence probabilities in the cell weighted by equilibrium prevalence (*Θ*):

2.1 $$\mathrm{zoonotic}\;\mathrm{hazard}\;\mathrm{at}\;{\mathrm{cell}}_{x}=\sum_{1}^{i}\left({\mathrm{presence}}_{i}{\times \Theta }_{i}\right).$$

#### (iii) Comparing the impact of three scenarios SSP1, SSP4 and SSP5

As a baseline, the sustainable scenario in the near future (2025) was used comparing the hazard against different land-use change scenarios in 2050. The impact of potential fossil-fuelled future scenarios was assessed by comparing the zoonotic hazards projected in 2050 against the zoonotic hazard in the sustainable scenario in 2025 (SSP5–SSP1). Likewise, we compared the zoonotic hazard of a deepening inequality scenario in 2050 with the hazard of the sustainable scenario in 2025 (SSP4–SSP1). We subtracted the hazard on the sustainable pathway in 2050 from the hazard on the pathways SSP4 and SSP5 in 2050 to assess the avoided risk by following the sustainable scenario. The resulting value indicates the extent of hazard that may increase or reduce by not following the pathway leading to a sustainable scenario (SSP4 2050 versus SSP1 2050 and SSP5 2050 versus SSP1 2050). To assess the impact of different economies on zoonotic hazard, countries were grouped following the World Bank classification system: low, lower-middle, upper-middle, and high-income economies updated to 2021 [52]. SSP4 assumes differences between the rich and poor economies in their response to the demand for energy and food, thus large changes in hazard were expected.

All analyses were performed in R software using the R packages: rgbif [53], CoordinateCleaner [54], rgdal [55], sp [56,57], sf [58], raster [59] and rpart [60]. All the R scripts to reproduce the work are available in our repository here: https://doi.org/10.5281/zenodo.5062634.

## 3. Results

### (a) Hotspots and spatial distribution of zoonotic hazard

**Figure 2.**
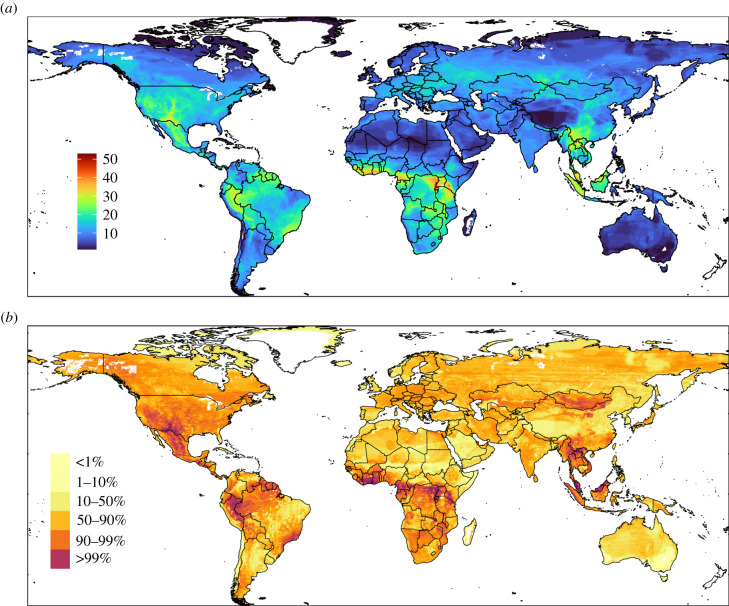
(*a*) Map of rodent-species richness according to data retrieved from GBIF and analysed based on land use according to CART analysis. (*b*) Hazard index predicted in the near future (2025) considering the sustainable scenario (SSP1-RCP 2.6). Legend values are showing percentiles (1, 10, 50, 90 and 99%).

The analyses herein revealed several hotspots of zoonotic hazard across the world (figure 2*b*). Most hotspots corresponded to locations with high diversity of rodent species (figure 2*a*), such as Southeast Asia, Southeast China, tropical areas of Africa and South America, and South-Central USA. Other areas with a high hazard index were Mongolia, some locations in Kazakhstan, Western and Eastern Europe, southern Africa and southern Argentina.

### (b) Zoonotic hazard on the shared socioeconomic pathways

#### (i) Hazards on the sustainable pathway (SSP1)

**Figure 3.**
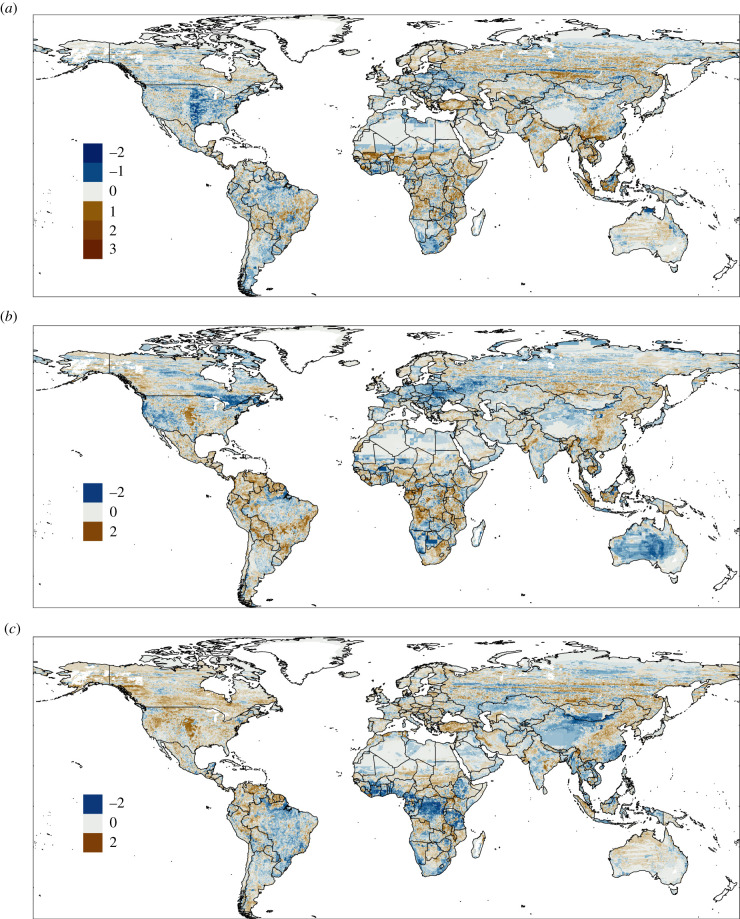
Hazard change from 2025 to 2050. Difference in hazard index predicted in three future scenarios, using the hazard index of 2025 as baseline: (*a*) SSP1 2050–SSP1 2025, (*b*) SSP4 2050–SSP1 2025, and (*c*) SSP5 2050–SSP1 2025. Positive values indicate an increase in the hazard index.

Globally, the hazard on the sustainable pathway SSP1-RCP 2.6 showed important changes by 2050 at the regional level from the hazard in our baseline map of 2025 (figure 3*a*). Regarding hotspots mentioned above, some of them demonstrated a clear reduction of zoonotic hazards, particularly the Amazon basin, southern Argentina, Mongolia and a large portion of Eastern Europe. Hotspot areas in Central Africa also evidence a decrease in several areas, but not as pronounced as the previous one. By contrast, South–Central USA, northern Mexico, southeast Brazil, Southeast China and Southeast Asia had a more heterogeneous response, with a mosaic of areas with different increments and reduction of zoonotic hazard.

Other relevant areas showing a reduction in zoonotic hazards are midwest, northeast, southeast USA and southeastern Africa. By contrast, an increase in hazards was located in some regions of Africa (sub-Saharan Sahel transmissive zone, central, west and east), Scandinavian Peninsula, Russia, Central America, Bolivia and southeastern Brazil (figure 3*a*).

#### (ii) Hazard on the pathway of deepening inequality (SSP4)

Compared with the sustainable pathway (SSP1), hazard in 2050 distributed differently on the SSP4 pathway (figure 3*b*). For example, hazards in hotspots in the American continent generally differed from the previous comparisons (SSP1 2050 versus SSP1 2025). Here, there was a general reduction in all western regions in the USA, but hazard increased across Mexico, Central America, northern South America and southern Argentina. Hotspots in Western and Eastern Europe showed a clear reduction in hazard, whereas hotspots in Africa and Southeast Asia showed similar patterns compared with hazards on the sustainable pathway.

Other areas that highly increased zoonotic hazards were South Africa, southwest Canada and a large portion located in the centre of the USA. By contrast, Australia generally decreased in hazard, which differed from the results of the sustainable pathway (figure 3*a*). In Europe, hazard decreased in general but increased in the Scandinavian Peninsula, the Mediterranean shore and Great Britain. Russia also would benefit from a global decline in hazard.

#### (iii) Hazard on the fossil-fuelled pathway (SSP5)

When comparing against the fossil-fuelled scenario, these results suggested an increase in hazard in large portions of the world globally (figure 3*c*). For example, in most hotspots and all other areas of North America, there was a marked increase in hazard compared with the other two scenarios (SSP1 and SSP4). Northern South America and southern Argentina generally increased their hazards, while southwest Brazil showed a decrease. Hotspots in Western and Eastern Europe demonstrated heterogeneity across their lands. There was a high reduction in hazard throughout the hotspots in Africa, while larger areas in Southeast Asia decreased their hazards, which differed from the other scenarios. Mongolia showed mixed results, with increasing and reducing hazards in the northern and southern parts, respectively.

Other areas with a high increase in hazards were, for example, central and eastern Russia and east-northern and central regions of China. By contrast, extensive areas of central Brazil and Northwest China hazard decreased.

#### (iv) Assessing the consequences of unsustainable pathways

**Figure 4.**
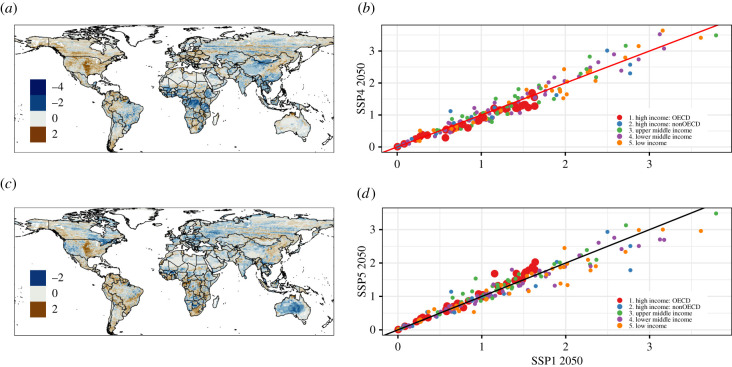
Hazard index predicted for 2050. (*a*) The pathway deepening inequality scenario 2050 compared with the sustainable future scenario 2050 (SSP4–SSP1). (*b*) Scatter plot of the hazard index according to countries classified by five classes of economies. Circles above the black line indicate an increase in hazard in SSP4, while circles below the black line indicate an increase in hazard in SSP1. (*c*) The fossil-fuelled development scenario 2050 compared with the sustainable future scenario 2050 (SSP5–SSP1). (*d*) Scatter plot of the hazard index according to countries classified by five classes of economies. Circles above the black line indicate an increase in hazard in SSP5, while circles below the black line indicate an increase in hazard in SSP1. In both scatter plots, countries are corrected per area.

The potential increase in zoonotic hazard of not abiding to sustainable development was assessed by subtracting the hazard forecasted for the sustainable scenario in 2050 from the hazard forecasted for the SSP4 and the SSP5 scenarios in 2050 (see Material and methods). The results are shown in figure 4.

In general, there was high variability across the world (figure 4*a*,*c*). The SSP4 scenario would decrease zoonotic hazard in some hotspots of rodent richness such as Central Africa, Southeast Asia and Southeast China (figure 4*a*). By contrast, hazard may increase in areas such as southern Argentina, northern South America and Eastern Europe. In the SSP5 scenario, zoonotic hazard in Western and Eastern Europe would remarkably decrease, while a mix of local outcomes (increase and decrease) is predicted in North America, Central Africa and Southeast Asia (figure 4*c*).

Changes in hazard have high variation among countries and regions, but the global change in zoonotic hazard seems centred at 0 on average (figure 4*b*,*d*). Variation is revealed when accounting for global economies. In several high Organisation for Economic Cooperation and Development (OECD)-income countries, hazard index increased under the SSP1 scenario compared with the SSP4 scenario (figure 4*b*). In addition, the maximum hazard change in these high-income countries would be relatively low (1.7) compared with the rest of the countries (low, medium and high non-OECD economies) that have variable hazard change, ranging from 0 to 3.6 (figure 4*b*,*d*). Under the SSP5 scenario, various high OECD-income countries increase their hazard under the SSP5 scenario compared with the SSP1 scenario (figure 4*d*).

## 4. Discussion

### (a) Hotspots and spatial distribution of zoonotic hazard

Our model describes the global spatial patterns of zoonotic hazard, accounting for rodent reservoir status and land-use change. This macroecological approach contributes to understanding global trends of wildlife reservoirs under different future scenarios. These analyses revealed several hotspots of zoonotic hazard in areas with high rodent-species richness [61]. This is in agreement with previous studies showing a link between mammal biodiversity and zoonotic hazard or disease risk [9,62,63]. Also, previous global studies highlight hotspot areas of disease emergence/risk in sites with high biodiversity and concurrently high degree of land-use change [3,23].

Several of the identified hotspots are current areas known to be endemic for rodent-borne diseases. For example, an important area of HPS [64] and Brazilian haemorrhagic fever (Sabia virus, family Arenaviridae) were identified [65] located in the highly fragmented Atlantic Forest in southeast Brazil. There are at least nine hantavirus strains and 16 rodent species associated with these hantaviruses in this region [66]. Furthermore, HPS risk in southeast Brazil is related to agricultural lands and forest fragmentation [16,64,67]. Several rodents are known to harbour hantavirus strains in North America, mainly in Mexico and the USA [68,69]. In Africa, some hotspots are concentrated in endemic areas of Lassa virus (family Arenaviridae) and monkeypox virus (family Poxviridae) [70,71]. Several microparasites transmitted by rodents, e.g. hantaviruses, *Leptospira* spp., *Bartonella* spp., *Trypanosoma* spp. and *Babesia* spp. are currently of public health concern in Southeast Asia [72].

In addition, hotspots with intermediate rodent-species richness were identified. These represent areas where rodent species with high equilibrium prevalence co-occur given the land use. For example, Mongolia has seven species of rodents (*Alticola macrotis*, *Alticola barakshin*, *Alticola semicanus*, *Alticola tuvinicus*, *Microtus maximowiczii*, *Microtus mongolicus*, *Meriones unguiculatus*), out of 180 species, with high equilibrium prevalence [25]. Another area of interest corresponds to southern Argentina, where six species (*Abrothrix olivaceus*, *Abrothrix longipilis*, *Eligmodontia morgani*, *Eligmodontia typus*, *Oligoryzomys longicaudatus*, *Phyllotis xanthopygus*) are among the 50 rodent species with the highest equilibrium prevalence [25].

The global patterns of zoonotic hazard were similar to the global patterns of species richness; however, the zoonotic hazard index showed inter-regional and local differences when observed in more detail. Therefore, these differences suggest that land-use impacts should not be neglected when using species richness as an initial conjecture to understand zoonotic hazards.

### (b) Zoonotic hazard on the shared socioeconomic pathways

Globally, zoonotic hazards in the future scenario of fossil-fuelled development (SSP5) differ from the scenario of deepening inequality (SSP4). However, hotspots were detected that will have high zoonotic hazards, regardless of the scenario assumed in 2050. Remarkably, zoonotic hazards increased in a large hotspot across the mid and southwest regions of the USA, where several rodent species with very high equilibrium prevalence can co-occur (e.g. *Microtus pennsylvanicus*, *Neotoma micropus*, *Peromyscus attwateri*, *Peromyscus boylii*, *Reithrodontomys megalotis*). This area corresponds to a mix of forest and pasture intensively transformed into croplands [73]. In both scenarios, SSP4 and SPP5, land is transformed into croplands, increasing the hazard of rodent-borne diseases. However, in the SSP4 scenario, high-income economies like the USA are expected to reduce population growth and expansion of croplands as they supply their energy and food demands with efficient livestock production. This capacity translates into a reduction of hazard in the region, compared with the sustainable scenario (figure 3*b*), and also compared with the scenario for SSP5 (figure 3*c*). In the latter, crops increased into pasture and forest, leading to an expansion of the zoonotic hazard (figure 3*c*).

Likewise, in South America and Africa, the SSP4 and SSP5 have contrasting results. In the fossil-fuelled scenario of SSP5, the hazard increase in these regions compared with the sustainable scenario and the scenario of deepening inequalities (SSP4). The narrative in SSP5 entailed a globalized economy, with the expansion of cropland reduced by open borders and international trade. In this scenario, middle and low-income economies could have a more limited expansion of croplands supplying their food and energy demands with the international trade. As in the SSP4 scenarios, borders are closed, inequalities emerged and developing countries increased crop expansion to fulfil their needs.

The increase in hazard in areas of high anthropogenic land-use agrees with recent studies that reported a general pattern at the local scale. In disturbed lands with intensive land-use change, rodent reservoir hosts tend to increase their abundance and richness, whereas non-reservoir hosts tend to decrease [10,30]. The unique ecology of rodent reservoirs may explain these results. In general, reservoir hosts live at a fast pace compared to non-reservoirs; they mature early, produce many offspring and have a short lifespan [31,34]. Being fast living and generalists, reservoir hosts can adapt to human-dominated habitats [74–78]. For instance, *Peromyscus leucopus* and *Peromyscus maniculatus* are reservoirs of several pathogens, e.g. *Babesia microti*, *Borrelia burgdorferi*, hantaviruses and are the most common rodents in cropland in the midwest USA [79]. Another example is the South American cricetid *Ab. olivaceus*, a rodent species with the highest equilibrium prevalence [25], which often predominates human-dominated landscapes [80,81].

A plausible explanation for the decrease in hazard in disturbed areas is that elevated disturbance levels, including considerable land-use change (e.g. forest to grassland for livestock), may also have depauperate faunas, i.e. defaunation [82]. Disturbance levels may be sufficiently strong to decrease the occurrence of a wide spectrum of rodent species, including both reservoir hosts and non-reservoir hosts. Furthermore, not all agricultural and forest plantations increase the abundance of reservoirs and the prevalence of rodent-borne zoonosis. For instance, the abundance of reservoirs of New World hantaviruses and the prevalence of these viruses in arid and temperate ecosystems are higher in natural vegetation than agricultural and timber plantation matrices [67,83].

### (c) Consequences of the unsustainable pathways

It is estimated that land-use change has impacted 32% of the global land area in the last six decades [84], and 56% of the less impacted surface is heavily fragmented in small patches and exposed to edge effects [85]. Our results demonstrated that land-use change could increase the hazard of zoonotic diseases as supported by previous studies [10,30]. Collateral effects of land-use development, particularly the emergence of infectious diseases, must be considered when estimating land-use societal or economic benefits.

Recent studies revealed strong links between vector-borne and zoonotic disease outbreaks by expanding livestock and agricultural lands at global scales [63,86], evidencing that unsustainable land-use development deteriorates human health, well-being and causes human suffering. If societies follow a sustainable path and restore degraded and depauperate ecosystems, a substantial reduction of diseases in humans might be possible [87]. For example, quantitative estimations show that reforesting the Atlantic Forest in Brazil will substantially reduce the hazard of 45% HPS cases, benefiting 3 million people [88].

However, a high variability within and among countries and regions was observed regarding the estimated zoonotic hazard change among scenarios. When comparing the sustainable scenario (SSP1) with the other two scenarios (SSP4 and SSP5), high-income countries had a lower degree of changes in the hazard index among scenarios. By contrast, medium- and low-income countries have more variation in their hazard projections. A sustainable pathway in low-income regions such as Central Africa and Southeast Asia would increase their zoonotic hazard index, which might be owing to the political and economic impacts discussed above.

Contrasting results between high- and low-income countries show no single global solution to reduce the effects of land-use changes on wildlife reservoirs and, therefore, on specific zoonoses. Monitoring and surveillance of biodiversity and ‘solution-oriented’ research agendas will help mitigate the hazard associated with land use [89,90]. The solutions to achieve a global impact appeared to be more local-regional rather than global.

### (d) Limitations of the study to infer disease risk

We acknowledge that our spatial analysis is restricted to a zoonotic hazard index derived from predicting the reservoir status of rodents [25]. Therefore, to account for human risk of rodent-borne pathogen exposure or risk of pathogen transmission is necessary to include different factors such as host density, rodent subspecies, pathogen circulation in a given area, pathogen prevalence, intermediate host populations, landscape structure, and human behaviour, among others [22,91,92]. All of these factors should be analysed at lower spatial scales (landscape-local). Also, our study only includes data on species occurrence and co-occurrence in a given area but does not incorporate species interactions, an important factor that can drive pathogen transmission. For example, species interactions may promote the dilution effect [9,93], observed in a variety of rodent-borne pathogens [72,94–98].

While our effort attempted to go beyond just presenting richness maps of co-occurring rodent hosts and future patterns of land-use change, we must be sober in interpreting the results. First, there is considerable uncertainty associated with land-use change models, which we tried to alleviate by presenting results under contrasting scenarios that capture possible variability. Second, these models are a global representation of potential futures and thus do not capture regional differences that could be more important.

## 5. Concluding remarks

Land-use change is thought to be a significant driver of the transmission and emergence of infectious diseases. Although the mechanisms by which diseases emerge are context and scale specific, there is an urgency to address the effects of environmental change on infectious diseases worldwide [99]. Conducting research using approaches from multiple spatial scales would be helpful to get a hold of these challenges. From a macroecological point of view, this study attempted to advance knowledge on the global effects of land-use change on the occurrence of rodents with implications for disease hazard. We anticipate that this information will aid in identifying geographical areas that should prioritize research and monitoring of rodent populations and their parasites, and rodent-borne diseases. Furthermore, the approach and methodology used in this work could also be useful in researching other wildlife reservoirs and zoonotic diseases.

Regardless of the pathway taken or the affected country, the risk of rodent-borne diseases could be a permanent threat. However, low-income countries will probably have to shoulder the burden of these diseases in terms of health and economy. By contrast, high-income countries will probably be better prepared and proactively respond to this threat. There are no simple solutions to prevent the emergence of infectious diseases. Transformative change and nature-based solutions that provide better use of the planet's natural resources, reducing the types of consumption, globalized agricultural expansion and trade may reduce biodiversity loss, ecosystem degradation and the risk of infectious diseases.

## Data accessibility

All data were obtained from publicly available datasets and indicated in the manuscript. All data and analysis are available in our repository: https://doi.org/10.5281/zenodo.5062634.
